# Inhibition of Bacterial Conjugation by Phage M13 and Its Protein g3p: Quantitative Analysis and Model

**DOI:** 10.1371/journal.pone.0019991

**Published:** 2011-05-26

**Authors:** Abraham Lin, Jose Jimenez, Julien Derr, Pedro Vera, Michael L. Manapat, Kevin M. Esvelt, Laura Villanueva, David R. Liu, Irene A. Chen

**Affiliations:** 1 FAS Center for Systems Biology, Harvard University, Cambridge, Massachusetts, United States of America; 2 Howard Hughes Medical Institute and Department of Chemistry and Chemical Biology, Harvard University, Cambridge, Massachusetts, United States of America; 3 School of Engineering and Applied Sciences, and Program for Evolutionary Dynamics, Harvard University, Cambridge, Massachusetts, United States of America; University of Birmingham, United Kingdom

## Abstract

Conjugation is the main mode of horizontal gene transfer that spreads antibiotic resistance among bacteria. Strategies for inhibiting conjugation may be useful for preserving the effectiveness of antibiotics and preventing the emergence of bacterial strains with multiple resistances. Filamentous bacteriophages were first observed to inhibit conjugation several decades ago. Here we investigate the mechanism of inhibition and find that the primary effect on conjugation is occlusion of the conjugative pilus by phage particles. This interaction is mediated primarily by phage coat protein g3p, and exogenous addition of the soluble fragment of g3p inhibited conjugation at low nanomolar concentrations. Our data are quantitatively consistent with a simple model in which association between the pili and phage particles or g3p prevents transmission of an F plasmid encoding tetracycline resistance. We also observe a decrease in the donor ability of infected cells, which is quantitatively consistent with a reduction in pili elaboration. Since many antibiotic-resistance factors confer susceptibility to phage infection through expression of conjugative pili (the receptor for filamentous phage), these results suggest that phage may be a source of soluble proteins that slow the spread of antibiotic resistance genes.

## Introduction

Widespread bacterial resistance to an antimicrobial agent typically occurs within three years of the introduction of a new antibiotic [1], [2]. Because mechanisms of resistance already exist for antibiotics derived from natural sources, horizontal gene transfer can rapidly transmit these mechanisms to pathogenic bacteria under selective pressure from the antibiotic. Conjugation is believed to be the major mechanism of transfer of antibiotic resistance genes [3], [4]. For example, individual cases of infection by vancomycin-resistant *S. aureus* are believed to have arisen from independent conjugation events within patients simultaneously colonized by vancomycin-resistant enterococcus and methicillin-resistant *S. aureus* [5]. The worldwide spread of extended-spectrum β-lactamases, particularly the widely distributed CTX-M-15 enzyme, is due to mobile genetic elements including conjugative plasmids from the IncF families (that encode F-like plasmids) [6]. Conjugative plasmids often carry resistance genes for multiple antibiotics from different classes, such that selection for resistance to one drug inadvertently selects for resistance to others [7]. For example, CTX-M-15 is carried on plasmids that also encode resistance genes against tetracycline and aminoglycosides [6]. In principle, inhibiting conjugation could potentially prolong the useful lifetime of antibiotics. For example, small-molecule inhibitors of enzymes involved in conjugative gene transfer may be useful in antimicrobial therapy [8].

Plasmids that enable bacterial conjugation encode a pilus that is expressed from the donor cell and binds to the recipient cell, mediating DNA transfer. Conjugation itself can occur between distantly related species, but some plasmids, such as the well-studied F plasmid, have a narrow host range (Enterobacteriaceae) due to incompatibilities of the replication system [9], [10]. Although the plasmid may carry genes for antibiotic resistance, the presence of a conjugative pilus can also confer a substantial disadvantage to the host cell, since the pilus is used as the site of attachment for certain DNA and RNA bacteriophages. In particular, the filamentous phages are a family of single-stranded DNA phages that attach to the tip of the conjugative pilus. The Ff family of phages (M13, fd, and f1) attach to the F pilus, which retracts and brings the phage into contact with the host cell coreceptor TolA, leading to transfer of the phage genome into the cell. Infectivity is mediated by the phage minor coat protein g3p, which contains three domains separated by glycine-rich linkers. In the initial step of phage infection, one N-terminal domain (N2) binds the tip of the F pilus, followed by the other N-terminal domain (N1) binding TolA, an integral membrane protein that confers colicin sensitivity. The C-terminal domain anchors the protein in the capsid and enables release of the phage particle from the host cell [11]. These phages establish a chronic infection that reduces the fitness of the host cell by 30–50% as phage particles are released continually [12].

Several decades ago M13 phage was observed to inhibit bacterial conjugation [13], [14], but the mechanism was unknown. Several possible explanations exist. First, phage reduce the fitness of cells and therefore may cause F+ cells to decrease in relative proportion over time, thereby eliminating conjugation donors. Second, infection appears to cause pilus retraction, so the F+ cells might not be competent as donors. Infection does reduce the average number of pili per cell, but infected cells still possess pili (from an average of 3.4 to 0.73 pili per cell) [15]. Third, the N-terminal domains of g3p may block contact with a recipient cell through physical occlusion of the F pilus or TolA. Overexpression of the N-terminal domains of g3p, which leads to periplasmic localization of the protein, was found to inhibit conjugation and infection by Ff phage, indicating that g3p plays an important role and that infection itself is not required for inhibition of conjugation [16]. Furthermore, although *tolA* mutants are resistant to colicin and filamentous phage infection, they do produce pili and conjugate normally [17], suggesting that interference with TolA is not the primary mechanism.

In this study, we demonstrate that non-replicating phage particles inhibit conjugation at similar concentrations to replicating phage, indicating that expression of phage genes is not necessary for phage-mediated inhibition of conjugation. Furthermore, exogenous addition of the soluble N-terminal domains of g3p (g3p-N), a minor coat protein of M13, results in nearly complete inhibition of conjugation at low nanomolar concentrations, suggesting that interaction with the F pilus is the primary mechanism of inhibition. Another effect, pili retraction upon infection, plays a lesser but measurable role. We present a quantitative model for conjugation in the presence of phage, in which a simple binding equilibrium between phage (or g3p-N) and F+ cells accurately describes the major effect of inhibition of conjugation.

## Results

### Conjugation without phage

**Figure 1:**
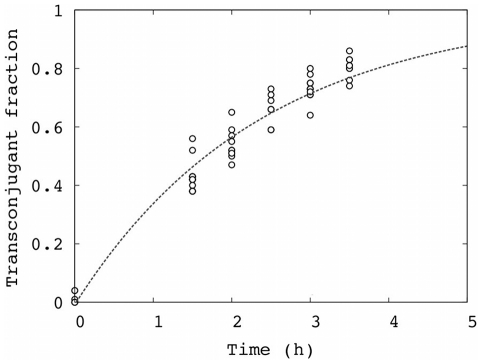
Conjugation without phage: Fraction of F+ cells that are transconjugants over time in the absence of phage. The line is a fit to the model of conjugation (*k_c_* = 0.42 /hr, *t_0_* = 0.02 hr, RMSD = 0.05). Data were pooled from several independent replicates.

To determine the rate of conjugation, we mixed F+ cells (TOP10F′; F′[lacI^q^ Tn10(tet^R^)]) expressing CFP (cyan fluorescent protein) with a large excess of F− cells (TOP10) expressing eYFP (enhanced yellow fluorescent protein). We followed the appearance of transconjugants over time during exponential growth in liquid culture as the transconjugant fraction (i.e., fraction of F+ cells exhibiting yellow fluorescence). Without phage, this fraction began at 0 after mixing and approached 1 during the course of the experiment due to the large excess of F− cells (Figure 1).

The simplest model that fits the data contains two parameters, growth rate (*k_g_*) and conjugation rate (*k_c_*), based on the mass-action model by Levin et al. [18], and can be described by the following reactions:
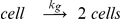


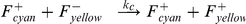


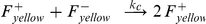
In this model, we assume that the growth rate of all cells is identical. We measured growth rates of F+ and F− cells in liquid media and found that there is only a slight difference, if any (Table 1). In our experiments, the high concentration of F− cells also permits the assumption that conjugation is zeroth-order with respect to F− cells [19]. This results in the following expression for the transconjugant fraction over time (*t*) (Text S1):
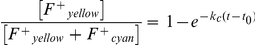
The growth rate does not appear in this equation. The first-order rate constant *k_c_* can be determined by fitting the data to this form (Figure 1), resulting in an estimate of *k_c_* = 0.42 /hr in the absence of phage (*t_0_* = 0.02 hr).

**Table 1:** Exponential growth rates of cells in liquid media.

Cell type	Fluorescence	Phage or protein	*k_g_* (hr^−1^)
F+	cyan	none	1.09±0.03
F−	yellow	none	1.18±0.11
F+	cyan	M13-km^R^	0.74±0.07
F+	cyan	M13-km^R^	* 0.73±0.09
F+	yellow	none	1.09±0.11
F+	cyan	100 nM g3p	1.11±0.09
F−	yellow	100 nM g3p	1.25±0.20
F+	yellow	100 nM g3p	1.13±0.06

Growth rate was measured by OD over time, except in row four (*) in which tetracycline-resistant colony counts were taken over time during conjugation experiments.

### Inhibition of conjugation by replicating M13 phage

**Figure 2:**
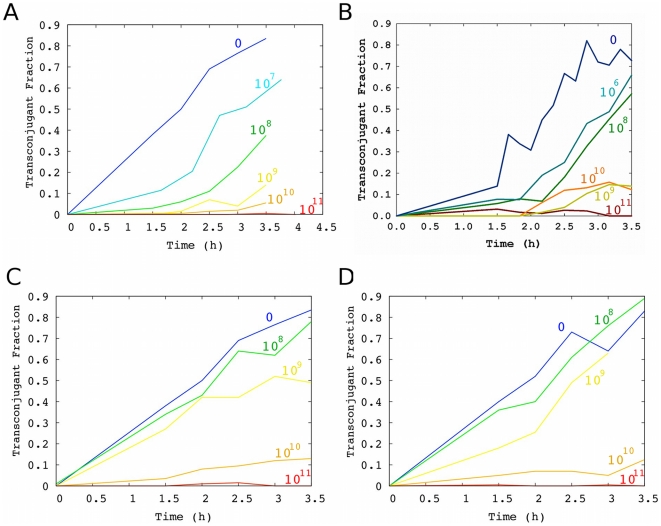
Transconjugant fraction over time with varying levels of phage or phagemid particles. For visual clarity, each line represents a single experiment, although multiple experiments were performed to accurately determine the conjugation rate. **Inhibition of conjugation by replicating M13 phage**: (A) M13-km^R^ (dark blue = not infected; for pre-infected F+ cells, light blue = 10^7^ pfu/mL, green = 10^8^ pfu/mL, yellow = 10^9^ pfu/mL, orange = 10^10^ pfu/mL, red = 10^11^ pfu/mL); (B) M13-amp^R^ (dark blue = not infected; for pre-infected F+ cells, light blue = 10^6^pfu/mL, green = 10^8^ pfu/mL, yellow = 10^9^ pfu/mL, orange = 10^10^ pfu/mL, red = 10^11^ pfu/mL); (C) M13-km^R^ (F+ cells were not pre-infected; blue = not infected, green = 10^8^ pfu/mL, yellow = 10^9^ pfu/mL, orange = 10^10^ pfu/mL, red = 10^11^ pfu/mL). (D) **Inhibition of conjugation by non-replicating phagemid particles lacking phage genes**: phagemid particles (blue = no phagemid particles added, green = 10^8^ cfu/mL, yellow = 10^9^ cfu/mL, orange = 10^10^ cfu/mL, red = 10^11^ cfu/mL).

We modified the commercially available phage M13KE by inserting a kanamycin resistance cassette into the multiple cloning site, thus enabling selection for infected cells when desired. As expected, cells infected with M13-km^R^ exhibited a slower growth rate (Table 1). To verify that M13-km^R^ inhibits conjugation, we grew a culture of F+ cells infected by M13-km^R^ under selection for kanamycin resistance to prevent loss of infection (donor cells). We further added varying amounts of purified M13-km^R^ phage to the media at the beginning of the conjugation experiment. We found that infected F+ cells conjugated with slightly reduced but still good efficiency at low levels of externally added phage (e.g., 10^7^ particles/mL), but the conjugation rate decreased as the phage concentration increased, such that almost no transconjugants were detected if the concentration of phage was ≥10^11^ particles/mL (Figure 2A). To check that this effect was not dependent on the specific plasmids carrying fluorescent markers or on kanamycin resistance, we also performed these experiments with F+ and F− cells carrying marker plasmids based on pRG5, with the F+ cells infected by M13-amp^R^. Similar results were obtained in this alternative system (Figure 2B).

The observation that infected cells were still competent donor cells suggested that most of the inhibition of conjugation is due to the external addition of phage rather than the effects of phage on the physiology of infected host cells. However, infected cells were also somewhat poorer donors than uninfected cells (compare Figure 2A and 2C; see Results: Model of inhibition of conjugation by replicating phage). We further verified that external addition of M13-km^R^ to initially uninfected cells also nearly totally inhibited conjugation at high phage concentrations (Figure 2C).

### Inhibition of conjugation by non-replicating phagemid particles lacking phage genes

If expression of phage genes (e.g., g3p) in the host cell is not the primary cause of reduced conjugation, then we expect that phage capsids bearing g3p but encapsulating a non-replicating phagemid lacking phage genes would also inhibit conjugation. We assayed conjugation in the presence of varying concentrations of the non-replicating phage (see Methods). We found that these particles inhibit conjugation to a similar extent as M13-km^R^, suggesting that a component of the phage capsid, rather than phage gene expression, is primarily responsible for inhibition (Figure 2D).

**Figure 3:**
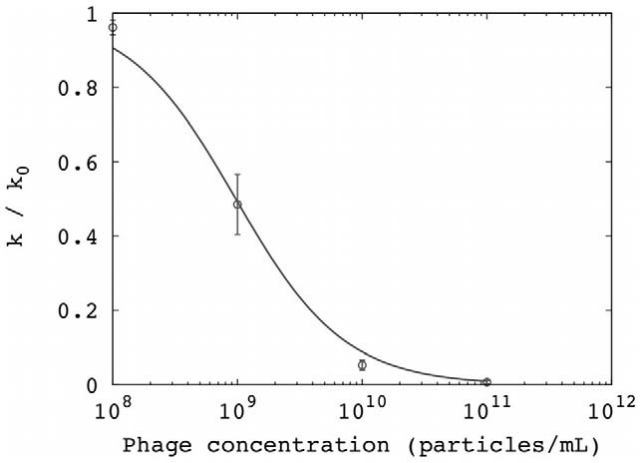
Inhibition of conjugation by non-replicating phagemid particles lacking phage genes: Inhibition curve for conjugation reflects the association between phagemid particles and F+ cells. The y-axis is the conjugation rate *k* normalized by the conjugation rate in the absence of phage (*k_0_*); error bars are standard deviation from replicates. The line represents the fit to a binding equilibrium (*K_d_* = 2 pM; RMSD = 0.03).

We hypothesized that the phagemid particles inhibit conjugation by simply binding to the tip of the F pilus and rendering them incapable of contacting potential recipient cells. We modeled this as a binding equilibrium between F+ cells and phagemid particles, with dissociation constant *K_d_*, in which the complex formed is incapable of conjugation (i.e., [phage][F+] = *K_d_* [complex]). This effectively reduces the concentration of competent donor F+ cells, such that the ratio α of the conjugation rate in the presence of phage particles (*k_c_^phage^*) to the basal conjugation rate (*k_c_*) is given by α = [F+]/([F+]+[complex]) = 1/(1+[phage]/*K_d_*). We fit the conjugation data in the presence of the phagemid particles to the simple model of conjugation and determined *k_c_^phage^* for several phage concentrations. The relationship between relative conjugation rate and phage concentration could be fit using the binding equilibrium (Figure 3), with apparent *K_d_* = 2 pM. This model could also be naively applied to conjugation kinetics in the presence of replicating phage (apparent *K_d_* = 2.4 pM; Figure S1). However, the concentration of phage increases over time due to infection and replication, so this procedure is likely to underestimate the true affinity of the interaction.

### Inhibition of conjugation by minor coat protein g3p from M13

**Figure 4:**
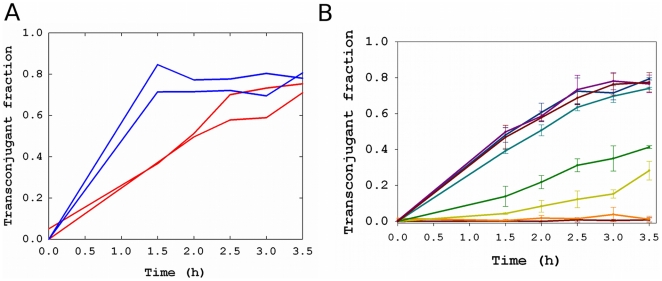
Inhibition of conjugation by minor coat protein g3p from M13. (A) Negative controls do not show inhibition of conjugation. Red line: BSA (100 nM). Blue line: chimeric phage (10^11^ pfu/mL). Replicates are shown. (B) Inhibition of conjugation by the soluble fragment of g3p. For visual clarity, each line represents one experiment, and error bars are standard deviation calculated from different platings in the same experiment. Multiple experiments were performed to estimate the conjugation rate. Dark purple = no protein; light purple = 0.1 nM g3p-N, dark blue = 0.5 nM, light blue = 1 nM, green = 5 nM, yellow = 10 nM, orange = 100 nM, red = 250 nM.

Because most of the inhibition of conjugation could be described well by a binding equilibrium between phage and host cell, we hypothesized that the major effect was caused by g3p, the N-terminal domains of which interact with the host receptor (F pilus) and co-receptor (TolA), while the C-terminal domain is a structural element of the phage coat [20]. To test the necessity of the N-terminal domains of g3p for inhibition of conjugation, we replaced these domains of M13-km^R^ with the homologous sequence from the related filamentous phage If1, which binds to I pili rather than F pili [21]. This experiment therefore created a functional knockout of g3p-N. The purified chimeric phage formed a clear stock solution in TBS buffer, suggesting that aggregation was minimal, and these phage also inhibited conjugation mediated by the I plasmid (Figure S2). As expected, the chimeric phage was not able to infect F+ cells, as assayed by transmission of kanamycin resistance, and the chimeric phage did not inhibit conjugation (Figure 4A).

**Figure 5:**
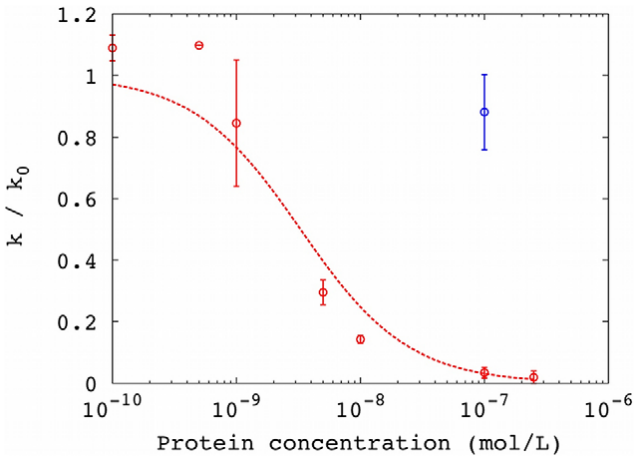
Inhibition of conjugation by minor coat protein g3p from M13: Inhibition curve for conjugation in the presence of g3p-N protein (red) or BSA (blue). Line represents model fit to a binding equilibrium (*K_d_* = 3 nM; RMSD = 0.1). Error bars indicate standard deviation from experimental replicates.

To confirm that g3p was the causative factor, we obtained a purified soluble fragment of g3p comprising the N-terminal domains (g3p-N) [22]. Addition of this protein to the media inhibited conjugation (Figure 4B), and the inhibition could be described well as a simple binding equilibrium between protein and F+ cells, similar to that described above for the non-replicating phage particles (Figure 5). The dissociation constant of this equilibrium was found to be 3 nM. The presence of g3p-N did not alter the growth rate of cells (Table 1). Addition of bovine serum albumin to high concentration did not appreciably inhibit conjugation (Figure 4A, Figure 5).

To test whether surface presentation of g3p would affect the conjugation rate, we bound g3p-N (His-tagged) to ion metal affinity chromatography beads. No difference in conjugation rate was observed at sub-saturating or saturating ratios of protein∶bead (Figure S3).

### Replication rate of phage

**Figure 6:**
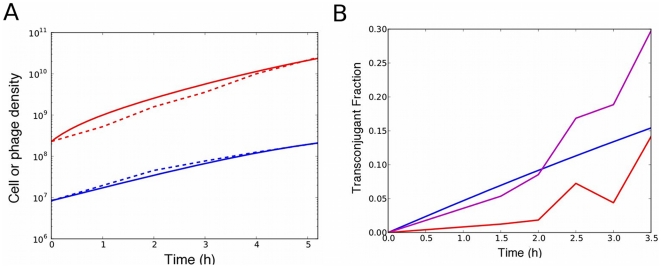
Model and experimental data for phage replication in F+ culture (all cells infected). (A) **Replication rate of phage.** The y-axis is the phage (red) or cell (blue) concentration over time. Experimental data are shown with the dotted lines; model fit is shown with the solid lines (*k_r_* = 60 /hr, RMSD = 3×10^9^ units/mL). (B) **Model of inhibition of conjugation by replicating phage**: Example of conjugation data (in this example, M13-km^R^ was added to 10^9^ pfu/mL; cells were pre-infected), with fit to determine *R* and *k_i_*. Data are shown in red and purple (two replicates), with model fit shown in blue (*R* = 0.2, *k_i_* = 10−8 /hr; RMSD over all conjugation trials = 0.32).

The conjugative ability of infected cells was qualitatively lower than that of non-infected cells (Figure 2A,C), especially at low phage concentrations. To quantify this difference, we first determined the replication rate of phage in order to model the phage concentration in the media throughout the experiment. We then fit the cell and phage concentration over time to a model of growth and replication, described by the following equations:
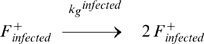


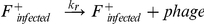
We determined *k_g_^infected^* from independent cell density measurements in liquid culture. Therefore, we fit the phage replication data with a single parameter *k_r_* to obtain *k_r_* = 60 /hr (Table 1; Figure 6A), i.e., about 60 phage particles are produced per hour by each infected cell.

Because this replication rate was somewhat lower than previously reported values of ≥100 /hr [23], [24], we checked whether our host strain produced a lower phage titer than less extensively modified strains (e.g., Top10F′ is a *recA*- strain). We grew an overnight culture of M13-amp^R^ using a relatively wild-type strain, *E. coli* ER2738, purified the phage, and obtained a plaque-forming titer of 2×10^11^ pfu/mL from the original culture. This also established the conversion factor of 2×10^12^ pfu/mL per A_270_ unit per cm. We repeated this experiment for M13-km^R^ and also found a concentration of 2×10^11^ pfu/mL of the original culture according to the absorbance measurement. To compare with *E. coli* TOP10F′ as the host strain, we measured plaque formation from a saturated overnight culture, which gave a titer of 5×10^10^ pfu/mL. We also measured phage concentration from a saturated culture by ELISA detecting the major coat protein g8p and found a concentration of 3×10^10^ particles/mL, indicating that the ELISA assay and plaque-forming titer give similar estimates. Therefore, the use of TOP10F′ appeared to decrease phage production by approximately 5-fold (perhaps due to its *recA1* genotype), possibly accounting for the reduced replication rate of our system.

### Model of inhibition of conjugation by replicating phage

Since we had independently determined the growth rates, effect of phagemid particles on conjugation rate and the replication rate of phage, there were only two unknown parameters when modeling conjugation with replicating phage: the infection rate of F+ cells by phage (*k_i_*) and the relative donor ability (*R*) of infected cells vs. uninfected cells. The relevant reactions are as follows, where *F^+^* refers to either yellow or cyan F+ cells, and α = [1/(1+[phage]/*K_d_*)] as before. Again, the concentration of F− cells in our experiments was sufficiently high that rate of conjugation was considered independent of [F−].
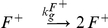


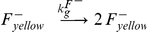


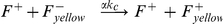


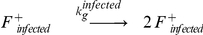


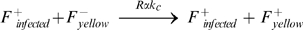


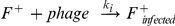


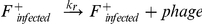
To determine *R* and *k_i_*, the data from multiple conjugation experiments in the presence of varying concentrations of M13-km^R^ were fit to this model (Figure 6B). The values that minimized the sum of squares of the deviations between model and experimental data were *R* = 0.2 and *k_i_* = 10^−8^ /hr. Because the experimental measurement of transconjugants over time is only an indirect measure of these parameters, we checked the robustness of the imputed values (see Text S1). The value of *R* was found to be robust to error, but *k_i_* was not, indicating that these data were not suitable to impute *k_i_*, and that the model fits were insensitive to changes in *k_i_*.

### Relative piliation of infected cells

To determine whether infected cells have reduced piliation in our system, perhaps accounting for a drop in donor ability upon M13 infection, we used fluorescently labeled phages specific for the F pilus (M13 and the RNA phage R17) to quantify the F pili associated with infected vs. uninfected cells. We found that infected F+ cells were characterized by approximately 2.5-fold less fluorescence than uninfected F+ cells, indicating reduced piliation.

### Test for loss of F factor

Although mechanisms exist to ensure proper segregation, the F factor can be lost at a low rate. Previous studies had indicated that F factor loss may be accelerated in the presence of filamentous phage [25]. To determine whether F factor loss occurred in our experiments, we measured the apparent growth rate of the original F+ cells during our conjugation experiments by counting cyan fluorescent colonies on media containing tetracycline (Table 1). If there had been substantial plasmid loss, the growth rate measured for tetracycline-resistant colonies would be lower than the growth rate measured by optical density in bulk solution. We found no significant difference between these measurements, suggesting that F factor loss did not occur to a substantial extent in our experiments.

## Discussion

The rapid increase in prevalence of bacterial pathogens that are resistant to multiple antibiotics has sparked investigation of new strategies for controlling infection. Conjugative plasmids have attracted attention as potential targets because of the high frequency of antibiotic resistance arising from conjugation, which can be >10^5^ times as frequent as spontaneous chromosomal mutation conferring resistance, and indeed resistance against β-lactams and aminoglycosides has been largely spread by conjugative transfer [26]. In addition, conjugative plasmids readily transmit multiple resistance genes. For example, *Klebsiella pneumoniae* EK105, a gram-negative pathogen that can cause refractory neonatal meningitis, carries resistance against 10 antibiotics on two plasmids (one conjugative and one mobilizable plasmid) [27]. Since conjugation can occur between closely related (e.g., within Enterobacteriaceae) or distantly related organisms (e.g., gram-positive to gram-negative), inhibiting conjugation has been suggested as a strategy to prevent or slow the spread of antibiotic resistance genes to dangerous pathogens [28]. Work in this direction has previously focused on inhibiting apparatus involved in DNA transfer [8]. In this work, we instead study a mechanism for blocking the formation of conjugal pairs. We expand upon a classic observation that filamentous phage inhibit conjugation and demonstrate that g3p, a coat protein of M13, can essentially completely block conjugation in liquid media.

We developed a simple mass-action model conjugation based on that of Levin et al. [18]. This simple model neglects two lags that are known to occur: 1) the donor cell takes some time to recover before it can act as a donor again, and 2) the transconjugant does not act as a donor cell immediately [29], [30]. Nevertheless, the model fit the kinetic data of transconjugant appearance well, suggesting that the simplifying assumptions of the mass-action model are reasonable within the experimental noise under these conditions. Under our experimental conditions of high F− cell concentration and exponential phase growth, the rate of conjugation could be assumed to be independent of F− (recipient) concentration. We further extended the mass-action model to include the effect of replicating and non-replicating bacteriophage M13 derivatives and minor coat protein g3p on conjugation and growth rate.

The parameters resulting from fitting our data appear to be reasonable given what is known about conjugation and infection. The apparent first-order conjugation rate constant of 0.42/hr was calculated from the simple mass action model applied to conjugation in the presence of a large excess of F− cells (>10^7^ cells/mL), without phage. Levin et al. determined a second-order conjugation rate constant of 2×10^−9^ mL/cell/hr for F-lac-pro exponentially growing cultures with cell densities in the order of magnitude of 10^8^ cells/mL, which corresponds to an apparent first-order rate constant of roughly 0.2 /hr [18]. Our assumption of apparent first-order kinetics that depend on F+ concentration but not F− concentration was based on the saturation phenomenon observed previously [19], in which the conjugation rate was found to be independent of recipient concentration at recipient densities >8×10^6^ cells/mL. The good fit to our data indicates this is a reasonable assumption.

To model experiments including replicating phage, we also determined the phage replication rate (*k_r_*) and the relative donor ability (*R*) of infected vs. non-infected cells. The replication rate *k_r_* was found to be 60/hr. This value is lower than that reported in the literature (≥100 /hr) for wild-type Ff phage [23], [24]. We believe this discrepancy is due in large part to the choice of host strain, since TOP10F′, a DH10B strain optimized for cloning, gave a 5-fold lower maximum titer than ER2738, a K12 *E. coli* strain used for phage propagation, thereby accounting for the difference between our measured replication rate and that reported previously. Another possible source of difference is the phage construct, since insertion of genes is likely to reduce efficiency of phage production.

We found that infection alone did indeed decrease donor ability by a factor of ∼5, independent of the external concentration of phage (*R* = 0.2). This effect quantitatively matches the effect that would be expected from the previously observed 4 to 5-fold decrease in F pili elaboration in infected cells (from an average of 3.4 to 0.73 pili per cell) [15]. We also quantified piliation in our system using fluorescently labeled F-specific phages (R17 or M13). This assay showed that infected Top10F′ cells have approximately 2.5-fold less pili than uninfected cells, in reasonable agreement with the observed effect on donor ability, although additional physiological factors reducing donor ability cannot be ruled out.

As resistance to antibiotics becomes more widespread among pathogenic bacteria, research into the potential medical utility of bacteriophages and their protein products is of particular interest. For example, a lytic enzyme isolated from a streptococcal phage can eliminate pneumococcal infection in a mouse model [31], and T7 phage expressing an enzyme that digests a component of extracellular polymeric substances, dispersin B, can effectively penetrate *E. coli* biofilms and lyse the cells contained within [32]. Filamentous phage in particular have been used recently to deliver genes that enhance the effect of antibiotics by blocking the SOS pathway in bacterial cells [33], and the addition of filamentous phage inhibits formation of bacterial biofilms due to interference with the formation of mating pairs [34]. The inhibition of conjugation by filamentous Ff phage was first observed several decades ago [13], but the dose-response curve and mechanism were not determined. Because conjugation is believed to be a major route of transfer of antibiotic resistance genes, we quantified the effect of phage M13 on conjugation and found that the mechanism of inhibition appears to be primarily a physical interaction between minor coat protein g3p and the host cell. This is particularly important at higher phage concentrations (i.e., above the *K_d_*, or >2 pM or 10^9^ particles/mL). At sufficiently high concentrations of phage, conjugation is essentially completely blocked.

An additional likely mechanism is the reduction in pili per cell after phage infection. This is in quantitative agreement with our observation that infection itself decreases donor ability by a factor of ∼5 (*R* = 0.2). Although this is a small contribution at high phage concentrations, it could be an important factor at low phage concentrations. In other words, at low levels of phage infection, the donor ability of the infected cells would be somewhat decreased but conjugation would continue. As infected cells secrete phage particles and the extracellular concentration approaches 10^9^ particles/mL, then conjugation would rapidly become nearly completely inhibited through occlusion of the F pili. Another possible mechanism of inhibition is the decreased fitness of infected F+ cells; if this fitness cost were high enough, the F+ cells would die out and thus stop conjugation. However, phage particles that transmit a phagemid that is incapable of replicating inside the host cells show a similar level of inhibition as M13-km^R^ phage, indicating that infection is not required for inhibition.

Finally, overexpression of the N-terminal domains of g3p in *E. coli* has been found to cause several membrane-related defects, including increased permeability, tolerance to colicins, and reduced conjugative ability [16]. We found that phage infection itself reduced the conjugation rate by a relatively small factor, suggesting that expression of g3p in its usual physiological context does not show the same phenotype as overexpression in isolation, possibly because g3p is normally sequestered by packaging into phage particles. In particular, the overexpressed N-terminal fragment of g3p is transported through the inner membrane to the periplasmic space, where it may interact with the F pilus, whereas full-length g3p is trapped in the membrane until it is packaged and released.

We hypothesized that g3p inhibited conjugation by physical occlusion since g3p is known to interact with the F pilus, and a soluble fragment of g3p delays infection by phage fd when added exogenously [35]. The N-terminal domains of g3p confer infectivity by binding to the host receptor (F pilus) and coreceptor (TolA) [20]. Indeed, exogenous addition of the soluble fragment of g3p comprising the N-terminal domains inhibited conjugation, while addition of a non-specific protein, BSA, did not.

The apparent *K_d_* of whole phage (2 pM) differed from the apparent *K_d_* of the soluble fragment of g3p (3 nM) by a factor of approximately 1000. One important distinction between the phage and g3p protein is that phage binding is essentially irreversible [36], likely due to events downstream of g3p binding, when the phage capsid fuses with the cell membrane and the phage genome is transferred into the cytoplasm of the host cell. Since *K_d_* reflects the balance between the binding and dissociation reactions, the very low reversibility of phage binding could account for the large difference between phage and soluble protein. Another contributing factor could be avidity through cooperativity among several g3p molecules in the same capsid, since each phage particle contains 3–5 copies of g3p in close proximity at one end of the filament [37]. We attempted to mimic an avidity effect using beads saturated with immobilized g3p-N, but this presentation did not affect the conjugation rate. Since the geometry of phage-bound g3p is not necessarily correctly modeled by bead-bound g3p, this result does not exclude the possibility that avidity may be an important effect. Finally, a technical possibility is that the purified soluble fragment of g3p differs in conformation from g3p in its native context. However, this fragment of g3p has been previously crystallized and found to be structurally similar to homologous proteins from other filamentous phage [22].

We have demonstrated that conjugation mediated by the F factor can be effectively inhibited by exogenous addition of nanomolar concentrations of a soluble protein derived from M13, and by picomolar concentrations of a non-replicating phage. This result suggests that the filamentous bacteriophages that target the conjugative pili may be a source of candidate biomolecules for slowing the spread of antibiotic resistance genes. A large proportion of conjugative resistance (R) factors from natural isolates are related to the F plasmid (i.e., *fi+* plasmids), and the F-specific phages infect many strains bearing R factors [38]. As with the F factor, infection by M13 has been observed to lead to loss of an R factor in the cell population [25], [39].

While the F factor is the most well-studied conjugative system, others exist and can be responsible for the dissemination of medically important resistances [3]. More work is required to determine if this strategy could be applied in a realistic setting and whether it would be possible to extend this strategy to cover the most common conjugative systems. This strategy does present challenges. For example, cells may shed F pili into the media, requiring additional phage to bind free pili. The severity of this problem would presumably depend on the environmental conditions as well as the host strain. As with any negative selective pressure, cells may evolve to resist the inhibition of conjugation. Indeed, one advantage of g3p and phage proteins in general is that, in contrast to small organic molecules, a large number of variants could be readily evolved or engineered in the laboratory, potentially countering bacterial evolution. Another possible challenge is that conjugation may occur in environments or bacterial life-cycle stages that are not easily accessible to therapeutic intervention, although some important scenarios may be appropriate targets. For example, genotyping of R factors in two outbreaks of β-lactam resistant infections in the same burn unit was highly suggestive of conjugative transfer of R factor from *Pseudomonas aeruginosa* to *Klebsiella aerogenes* within a patient simultaneously harboring both organisms in his wounds [40]; such wounds might present an opportunity for conjugation inhibitors to curb antibiotic-resistant outbreaks. On the other hand, granulomatous infections might be inaccessible to similar therapy. Finally, other mechanisms for gene transfer may compensate for reduced conjugation, limiting the utility of this strategy. Nevertheless, the inhibition of bacterial conjugation may be worthy of further investigation as the use of antibiotics continues to favor the acquisition of resistance genes by pathogenic bacteria.

## Methods

### Bacterial strains, culture conditions, phage propagation and titering (Table 2)

**Table 2:** Strains, plasmids, and phages used in this study.

Type	Name	Source	Notes
Bacterial strain	*E. coli* TOP10F′	Invitrogen	Donor strain, F+
	*E. coli* TOP10	Invitrogen	Recipient strain, F−
	*E. coli* TG1	Stratagene	Phage cloning, protein production
	*E. coli* ER2738	New England Biolabs	Phage propagation
	*E. coli* DH10B/pir116	This work	Production of non-replicating phage
	*E. coli* ATCC 27065	ATCC	I+ strain
Plasmid	pRG5	D. Lovley [43]	Marker plasmid
	pRG5_gfp	D. Lovley [43]	Fluorescent marker plasmid
	pTrc99A-eYFP	H. Berg	Fluorescent marker plasmid
	pTrc99A-eCFP	H. Berg	Fluorescent marker plasmid
	pET21(g3p-N)	This work [22], [45]	Protein expression
Phage	M13-amp^R^	This work	
	M13-km^R^	This work	
	Chimeric phage	This work	M13-km^R^ (If1-g3p-N) Δ(M13∼–g3p-N)
	HPdO	Unpublished; GenBank HQ440210	Helper phage with deleted f1 ori
	R17	P. M. Silverman [46]	F-specific RNA phage
Phagemid	pJC126b	Unpublished; GenBank HQ440211	*pir*-dependent replication

*E. coli* TOP10F′ and TOP10F (Invitrogen; *mcrA* Δ(*mrr*-*hsd*RMS-*mcrBC*) φ80*lacZ*ΔM15 Δ*lacX74 recA1 araD* 139 (*ara leu*) 7697 *galU galK rpsL* (StrR) *endA1 nupG*) were used as a donor (F^+^) or recipient (F^−^), respectively (F′ plasmid: [*lacI^q^*, Tn10(TetR)]). *E. coli* TG1 [41] (*supE hsd*Δ5 *thi* Δ(*lac*-*proAB*) F′(*traD36 proAB* ^+^ lacI^q^ *lacZ* ΔM15)) and *E. coli* ER2738 (New England Biolabs; F′*proA^+^B^+^ lacI^q^ Δ(lacZ)M15 zzf::Tn*10(Tet^R^)*/fhuA2 glnV Δ(lac-proAB) thi-1 Δ(hsdS-mcrB)5*) were used for cloning and phage propagation, respectively. DH10B with the pir116 gene replacing the proBA locus was used for R6K replication to produce non-replicating phagemid particles (helper phage/phagemid system). This strain was recombineered using the protocol of Datsenko and Wanner [42].

Standard protocols were used for common bacterial and phage-related procedures [41]. All strains were grown in LB medium on a regular basis. Medium 2×TY was used for conjugation experiments. When needed, media were supplemented with ampicillin or carbenicillin (100 µg/mL), tetracycline (12 µg/mL), kanamycin (50 µg/mL) and isopropyl-β-D-1-thiogalactopyranoside (IPTG) (1 mM).

Cells used for M13 phage propagation were cultured in 400 mL of LB and phages were recovered from the culture supernatant after removing cells debris by two successive precipitations with a 2.5 M NaCl/20% w/w PEG 8000 solution and resuspension in TBS buffer. In the case of R17 propagation, 250 ml of host cells *E. coli* TG1 were grown until they reached OD_600_ = 1 and were infected with 10^9^ pfu of phage; the culture was incubated overnight at 37 degrees. Lysates were generated by adding 1 ml of lysis solution (0.1 mg/ml lysozyme, 0.01 M EDTA, 1 M Tris-HCl pH 7.5) and chloroform 1% v/v and incubating for one hour at room temperature with agitation. Next, 1 µg/ml of DNaseI (Sigma) was added to the lysates and they were incubated for 1 h at 4°C. Cell debris was removed by two steps of centrifugation at 5000 and 20,000 rpm respectively, and R17 phages present in the supernatants were recovered as described for M13. Phage concentration was estimated by measuring the absorbance of the sample at 273 nm (assuming a conversion factor of 2×10^12^ pfu/mL per unit of absorbance per cm), and by infecting *E. coli* TOP10F′, TG1 or ER2738 for 1 h at 37°C using an appropriate dilution of the phage. After plating on selective media and incubating for >16 h at 37°C, the number of colony-forming units (cfu)/mL was determined. Alternatively, plaque-forming titers were determined following a standard protocol. Phage particles were stored at 4°C for up to several months.

### Plasmids, phagemids, and phage genomes used in this study (Table 2)

DNA was manipulated using standard methods [41]. Plasmids pRG5 (Spc^R^) and the variant pRG5_gfp encoding GFP under the control of a *Plac* promoter were a kind gift from D. Lovley's lab at the University of Massachussetts, Amherst [43]. Plasmids pTrc99A-eYFP and pTrc99A-eCFP (Amp^R^) encoding, respectively, eYFP (Q95M; yellow) and eCFP (A206K; cyan) were courtesy of H. Berg at Harvard University.

Phage genomes were constructed using M13KE (New England Biolabs) as a scaffold. M13KE-NotI, which has a *Not*I restriction site inserted in frame with the g3p gene between the N and C-terminal domains, was constructed using Stratagene QuikChange XLII kit for site-directed mutagenesis using primers NotIplus (5′-CCTCCTGTCAATGCGGCCGCAGGCTCTGGTGGTGG) and NotIminus (5′-CCACCACCAGAGCCTGCGGCCGCATTGACAGGAGG). M13-amp^R^ was prepared by inserting an ampicillin resistance cassette (*bla*) into M13-KE. The *bla* gene was PCR amplified using primers bla1 (5′-TAGCCCAAGCTTGATGAGTATTCAACATTTCCG) and bla2 (5′-TCGCTGAATTCTTGAGTAAACTTGGTCTGAC) and the vector pCyanscriptIIKS(-) (Stratagene) as a template for the reaction. The PCR product was digested using *Eco*RI/*Hin*dIII restriction enzymes and cloned in the same sites present in M13KE-NotI. M13-km^R^ was constructed under the same scheme using oligonucleotides HindIII_Km_F (5′-GACTACAAGCTTTATGAGCCATATTCAACGGGAAAC) and EcoRI_Kan_R (5′-GTAGTCGAATTCGTGTTACAACCAATTAACCAATTCTG), and the transposome EZ-Tn5 <KAN-2> (Epicentre) as a template. The gene fragment encoding the 250 amino acids of the g3p homolog protein from If1 phage (NC_001954) comprising A11 through T260 was cloned into the M13-km^R^ scaffold. The fragment was amplified by PCR using the dsDNA from If1 phage as a template and the oligonucleotides If5 (5′- AAGGTACCTTTCTTTACCCATGCAACTACAGACGC) and If3 (5′-AAGCGGCCGCCGTAACCGTATCAGTTATCAAAACAGC). The resulting 754 bp DNA fragment was digested using *Kpn*I and *Not*I restriction endonucleases and cloned into M13 digested with the same enzymes, generating a translation fusion between the N-terminal domain of the g3p homolog from If1 and the C-terminus of the same protein from M13.

### Non-replicating phagemid particles lacking phage genes (Table 2)

To generate these phages, we used a *pir+* bacterial strain transformed by a helper plasmid encoding all phage genes but lacking the f1 origin of replication and packaging signal. This strain was also transformed with the pJC126b phagemid, which utilizes the R6K and f1 origins of replication and carries genes for chloramphenicol resistance and YFP. The phagemid is therefore packaged into particles by a *pir+* strain but cannot propagate upon infection of a *pir-* host strain, such as TOP10F′. Helper phage HPdO is an engineered variant of VCSM13 (Stratagene) that lacks the original f1 phage origin and the packaging signal (GenBank accession number HQ440210). Phagemid pJC126b (GenBank accession number HQ440211) also contains the *pir*-dependent R6K origin in addition to the f1 origin, catR, and YFP. Both constructs were made by uracil excision cloning [44]. *E. coli* DH10B (*pir116 ΔproBA*) cells transformed with helper phage and phagemid pJC126b were used to produce viral particles that were unable to replicate in TOP10F′ cells. We purified these phagemid particles and verified that while the titer of purified stock on *pir*+ cells was 10^12^ chloramphenicol-resistant cfu/mL, no colonies formed on our TOP10F′-derived host cells at the tested dilutions of the stock titer. Therefore, the titer on TOP10F′ was more than 10^5^-fold less than the titer on *pir*+ cells, indicating a defect of greater than five orders of magnitude for replication on TOP10F′.

### Cloning, expression and purification of the N-terminal domains of g3p from M13

The protein comprising the N-terminal domains of g3p (g3p-N) from M13 was expressed following the protocol previously described by P. Holliger and collaborators [22], [45]. Gene synthesis, expression and purification were carried out by BioBasic Inc. (Canada). Briefly, the g3p fragment comprising A19 to A237 was fused at the N-terminus to the 21 amino acids corresponding to the PelB leader sequence for secretion to the periplasm, and at the C-terminus to a 6His tag for purification. The sequence of the engineered gene is given in Text S1. The DNA resulting from the gene synthesis was cloned using *Eco*RV restriction endonuclease into a variant of pUC18 plasmid, pUC57, for sequencing. The gene was then subcloned using *Nde*I and *Xho*I restriction sites into vector pET21, enabling expression of the gene fused to an ATG codon in a *Nde*I site included in the polylinker that harbors a strong RBS. This plasmid (Table 2) was transferred to *E. coli* TG1 cells and the protein was expressed and obtained from the bacterial periplasm by resuspension of cells into ice-cold 5 mM Mg_2_SO_4_ and stirring during 10 min on ice followed by centrifugation and recovery of the supernatant. The mixture of proteins was loaded into a Ni^2+^ affinity column and g3p-N was further purified by gel filtration. The quality of the sample was verified by BioBasic, Inc. SDS-PAGE analysis showed a single band, the protein was 98% pure by HPLC, and the correct molecular weight was found by mass spectrometry.

### Conjugation experiment

The rate of conjugation was estimated by co-culturing F^+^ (donor) and F^−^ (recipient) cells. Separate overnight cultures of TOP10F′ (transformed by pTrc99A-eCFP) and Top10 (transformed by pTrc99A-eYFP) were inoculated in 3 mL of LB with ampicillin, IPTG, and tetracycline if appropriate. The fluorescent markers were expressed from amp^R^ selectable plasmids and facilitated identification of new transconjugants, which would be tet^R^ and yellow fluorescent. For experiments using pre-infected F^+^ cells, the overnight culture was inoculated from an infected colony grown on media with the appropriate antibiotic to select for phage (carbenicillin or kanamycin), and the overnight culture was also grown with this antibiotic in the media. The next morning, 30 µl of the overnight culture was used to inoculate separate tubes containing 3 mL of 2×TY with ampicillin and IPTG. Cells were grown at 37°C and 200–250 rpm until they reached OD_600_ of 0.2–0.3, determined using an Ultrospec cell density meter (GE Healthcare). The amount of phage added to the conjugation experiment due to addition of the infected F+ culture alone was ∼10^6^ particles/mL, as determined by ELISA assay on the supernatant. The ELISA assay gave quantitatively similar results to a plaque-forming assay for M13-km^R^ (see Results: Replication rate of phage). The number of physical particles, as opposed to plaque- or colony-forming units, appeared to be the most important contributor to the inhibition of conjugation (see Results: Inhibition of conjugation by non-replicating phagemid particles lacking phage genes).

The conjugation experiment was initiated by diluting the exponentially growing F^+^ cells by a factor of 100 in 2×TY and adding 150 µl of this preparation to the 3 mL of exponentially growing F^−^ cells at OD 0.2–0.3 (1∶2000 ratio of donor∶recipient). Phage particles, protein stock or beads coated with protein were also added at this point if desired. Coated beads were prepared as follows: Dynabeads His-Tag Isolation&Pulldown (Invitrogen) were washed with TBS buffer and then incubated with the appropriate amount of protein for 10 min at RT in the same buffer supplemented with 0.6 M NaCl. Binding of the proteins to the beads was confirmed by SDS-PAGE. The mixture was vortexed briefly and incubated at 37°C at 200 rpm. We verified that cultures grown under these conditions for 3.5 hours remained in exponential phase throughout the experiment by following the OD_600_ in a pilot experiment. Aliquots were removed from this mixture at different times up to 3.5 hours, diluted appropriately, and plated into H-agar medium supplemented with tetracycline, carbenicillin and IPTG, to minimize the effect of plate-to-plate variation in absolute colony counting. After an overnight incubation, the plates were scanned on a Typhoon TRIO variable-mode imager (Piscataway, NJ) using cyan laser excitation and detection at 488 nm. The colonies on each plate were either high or low fluorescence, corresponding to yellow and cyan fluorescence respectively, and the counts were recorded. When using cells transformed with pRG5 or pRG5_gfp, fluorescent colonies were detected on a Dark Reader (Clare Chemical Research, Inc., Dolores, CO).

### Enzyme-linked immunosorbent assay (ELISA) for phage

Phage concentration was determined using the Phage Titration ELISA kit (Progen) as indicated by manufacturer. Briefly, the phage concentration in a sample was determined by binding them to ELISA plates coated with a peroxidase-conjugated monoclonal antibody that specifically recognizes the major coat protein g8p. After binding and washing, wells were developed by adding hydrogen peroxide and the substrate tetramethyl benzidine (TMB) and the amount of phages is estimated by registering the A_450_ of the sample. A standard curve was generated using kit control sample. Test samples included supernatants or phage stocks diluted in TBS.

### Phage replication in F+ cells

To determine the replication rate of phage in infected F+ cells, we grew a culture of F+ cells infected by M13-km^R^ and measured the cell density over time by OD_600_. We also measured the phage concentration over time by removing an aliquot of the culture, pelleting the cells, and analyzing the supernatant by ELISA detecting the major coat protein of M13. F+ cells were inoculated as given for the conjugation protocol. If the OD_600_>1, the culture was diluted with media appropriately to bring the density into the linear range of the cell density meter. Aliquots were removed at time points and the cells were spun down, and the supernatant was stored on ice until the end of the experiment, when ELISA was performed on all the supernatant samples. An analogous experiment was performed with a 1∶2000 mixture of F+ to F− cells to simulate the conditions of a conjugation experiment. Data from this second experiment were used in combination with the conjugation data to estimate *R* and *k_i_*.

### Preparation of fluorescent phages

Fluorescent M13 and R17 phages were prepared using a modified version of a protocol described in a previous work [46]. Typically, 0.5–1 ml of phages around 10^12^ pfu/ml were dialyzed for 4 h at 4°C against a solution containing 0.1 M NaHCO_3_ (pH 8.5)/1 mM MgCl_2_. The solution was changed once and left overnight at 4°C. Alexa 488 carboxylic acid (succinimidyl ester; Invitrogen) (1 mg) was dissolved in 0.1 ml of anhydrous DMSO and stored in 10 µl aliquots. One aliquot was added to each of the phage preparations and the mixtures were incubated for 1 hour at room temperature with agitation. After that, phages were recovered and washed by two successive steps of precipitation with PEG/NaCl as described previously.

### Fluorescent phage binding and fluorescence measurements

Cultures (cyan F−, cyan F+ and cyan F+ preinfected with M13) were grown in LB media until mid-exponential phase, and 0.2 ml aliquots were incubated with 10 µl of fluorescent phages for 15 min at room temperature. Cells were recovered by centrifugation at 13,000 rpm and resuspended in 0.2 ml of M9 medium. The relative number of cells was estimated by measuring the constitutively produced CFP present in all cultures using an excitation wavelength of 444 nm and emission wavelength of 460 nm. Binding of Alexa 488 fluorescent phages was determined using an excitation wavelength of 485 nm and emission wavelength of 530 nm. Measurements from F− cells were used to correct for background autofluorescence. All fluorescence measurements were performed in a Spectramax Gemini XS plate reader (Molecular Devices). The relative piliation was calculated as the ratio between the signals obtained for Alexa 488 and cyan. Standard deviations were calculated from three independent experiments.

### Fitting models to experimental data

The differential equations corresponding to the chemical equations were solved by hand or by Mathematica to obtain the time evolution of the variables with the appropriate free parameters. The experimental data were fit by minimizing the sum of the squares of the deviation between the data and the model. More modeling details are given in the Text S1.

## Supporting Information

Figure S1 **Inhibition of conjugation by replicating M13 phage.** The simple binding model can be naively fit to the conjugation data for replicating phage (no pre-infection). The model fit is given by the line (*K_d_* = 2.4 pM). Experimental data points are shown with error bars corresponding to standard deviation among replicates. This model does not take into account phage replication and infection.
(TIFF)

Figure S2 **Inhibition of I-mediated conjugation by chimeric phage.** The number of tetracycline-resistant (I+) colonies was measured over time during conjugation of *E. coli* ATCC27065 and TOP10 in the presence (gray squares) or absence of chimeric phage (black circles). The chimeric phage was present at a concentration of 10^11^ pfu/ml.
(TIFF)

Figure S3 **Conjugation in the presence of immobilized g3p-N.** The transconjugant fraction was measured over time in the presence of free g3p-N (3.8 nM; yellow line), beads without g3p-N (red line), or in the presence of an equivalent amount of g3p-N bound at a sub-saturating density of 10^4^ molecules/bead (brown line) or at a saturating density of 10^5^ molecules/bead (blue line).
(TIFF)

Text S1 (DOC)
