# Supplementary figures and images for: Inhibition of Bacterial Conjugation by Phage M13 and Its Protein g3p: Quantitative Analysis and Model
Source: PLoS One. 2011 May 26;6(5):e19991. doi: 10.1371/journal.pone.0019991 (PMC3102678; doi:10.1371/journal.pone.0019991)

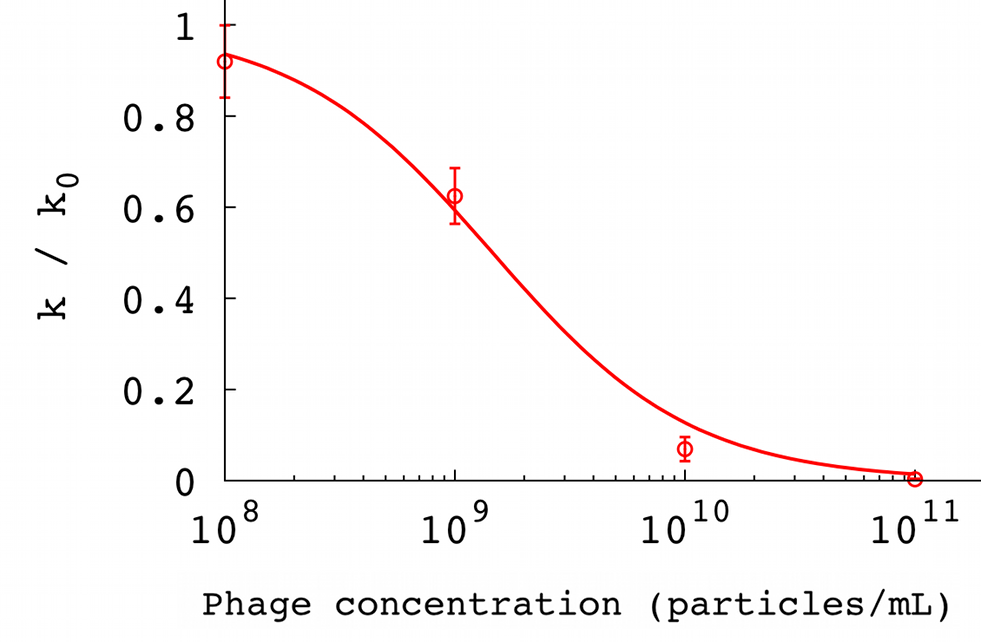

Supplement: Figure S1 — Inhibition of conjugation by replicating M13 phage. The simple binding model can be naively fit to the conjugation data for replicating phage (no pre-infection). The model fit is given by the line (Kd = 2.4 pM). Experimental data points are shown with error bars corresponding to standard deviation among replicates. This model does not take into account phage replication and infection. (TIFF) [file pone.0019991.s001.tiff]

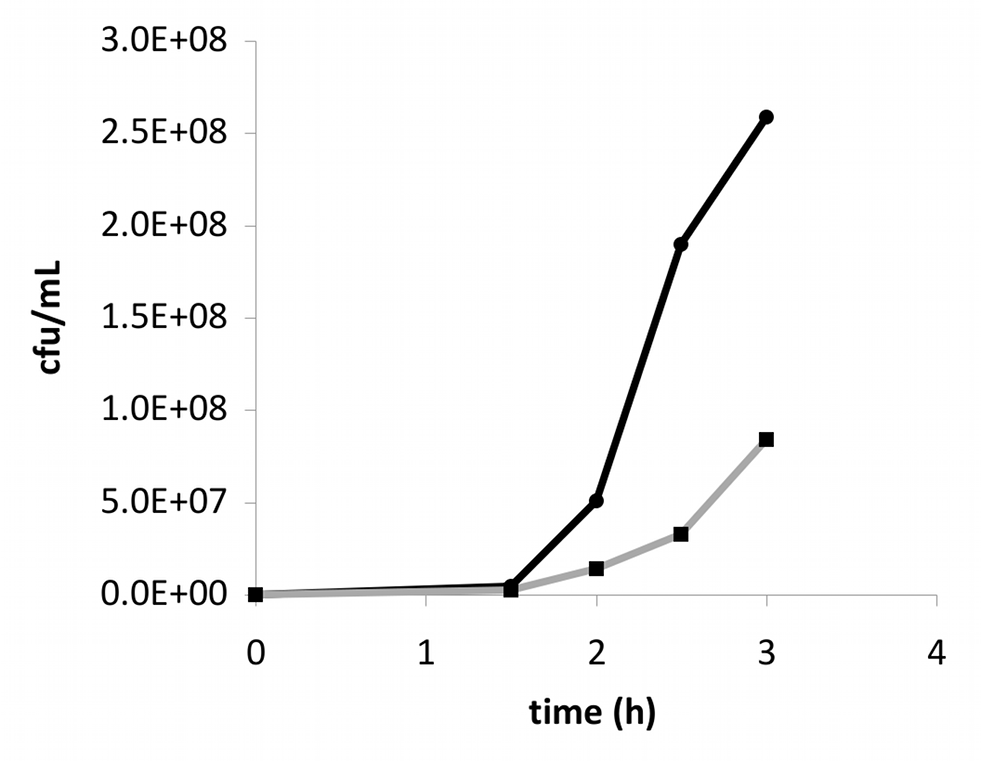

Supplement: Figure S2 — Inhibition of I-mediated conjugation by chimeric phage. The number of tetracycline-resistant (I+) colonies was measured over time during conjugation of E. coli ATCC27065 and TOP10 in the presence (gray squares) or absence of chimeric phage (black circles). The chimeric phage was present at a concentration of 1011 pfu/ml. (TIFF) [file pone.0019991.s002.tiff]

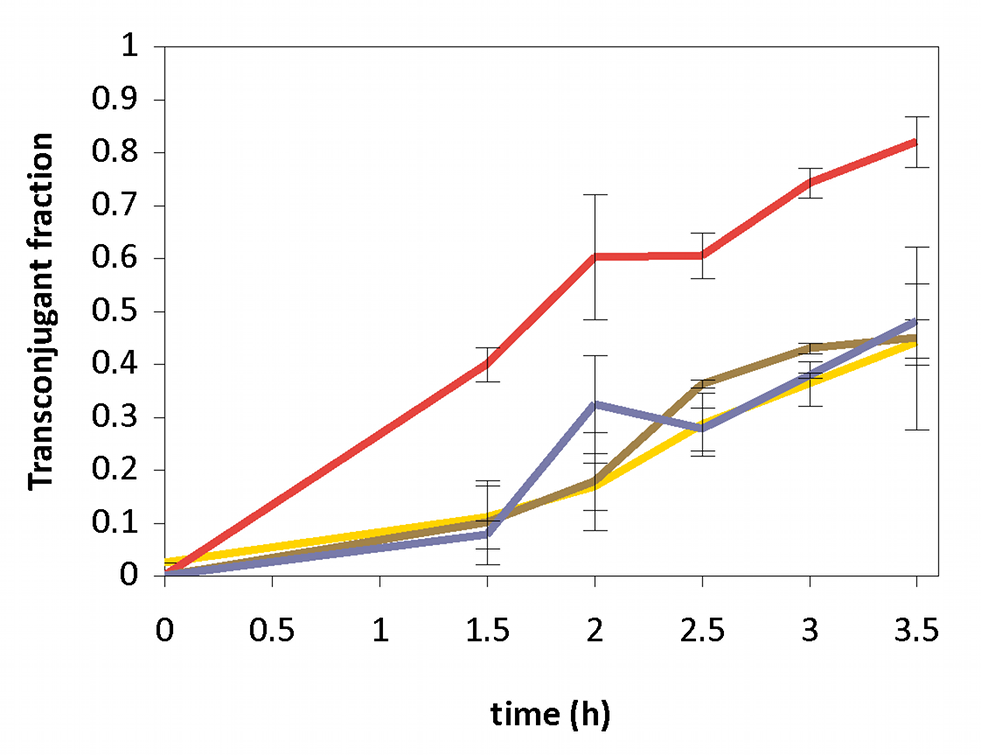

Supplement: Figure S3 — Conjugation in the presence of immobilized g3p-N. The transconjugant fraction was measured over time in the presence of free g3p-N (3.8 nM; yellow line), beads without g3p-N (red line), or in the presence of an equivalent amount of g3p-N bound at a sub-saturating density of 104 molecules/bead (brown line) or at a saturating density of 105 molecules/bead (blue line). (TIFF) [file pone.0019991.s003.tiff]
