# Supplementary material for: Inhibition of Bacterial Conjugation by Phage M13 and Its Protein g3p: Quantitative Analysis and Model
Source: PLoS One. 2011 May 26;6(5):e19991. doi: 10.1371/journal.pone.0019991 (PMC3102678; doi:10.1371/journal.pone.0019991)
Supplement: Text S1 — (DOC) [file pone.0019991.s004.doc]

**Inhibition of bacterial conjugation by phage M13:**

**quantitative analysis and model**

Abraham Lin, Jose Jimenez, Julien Derr, Pedro Vera, Michael L. Manapat, Kevin M. Esvelt, Laura Villanueva, David R. Liu, Irene A. Chen

**Text S1**

**DNA and amino acid sequence of g3p-N**

CATATGAAATACCTGCTGCCAACTGCGGCTGCTGGTCTGCTGCTGCTGGCAGCCCAGCCAGCAATGGCAGAAACGGTCGAAAGCTGTCTGGCGAAACCGCACACTGAAAACTCTTTTACCAACGTATGGAAAGACGACAAAACTCTGGACCGCTACGCGAATTATGAAGGTTGCCTGTGGAACGCTACTGGTGTGGTTGTTTGTACCGGCGACGAGACCCAGTGCTACGGCACTTGGGTGCCAATCGGCCTGGCGATTCCGGAAAACGAAGGCGGTGGTTCTGAAGGTGGCGGCAGCGAAGGTGGTGGTAGCGAGGGTGGCGGTACCAAACCGCCTGAATATGGTGACACCCCGATCCCGGGCTACACCTACATCAACCCGCTGGACGGCACCTACCCGCCGGGCACTGAACAGAATCCGGCAAACCCGAACCCGTCCCTGGAAGAATCCCAGCCGCTGAACACCTTCATGTTCCAGAACAACCGCTTCCGTAACCGTCAGGGCGCTCTGACGGTCTACACGGGTACCGTTACCCAGGGCACCGATCCGGTAAAGACCTACTATCAGTACACCCCGGTTTCCTCCAAAGCGATGTATGATGCCTATTGGAATGGTAAGTTCCGTGATTGCGCCTTCCACTCTGGTTTTAACGAGGATCCTTTCGTTTGCGAATACCAGGGTCAATCTTCTGATCTGCCGCAACCGCCGGTGAACGCGGCTGCACACCACCATCATCACCACTAATGACTCGAG

MKYLLPTAAAGLLLLAAQPAMAETVESCLAKPHTENSFTNVWKDDKTLDRYANYEGCLWNATGVVVCTGDETQCYGTWVPIGLAIPENEGGGSEGGGSEGGGSEGGGTKPPEYGDTPIPGYTYINPLDGTYPPGTEQNPANPNPSLEESQPLNTFMFQNNRFRNRQGALTVYTGTVTQGTDPVKTYYQYTPVSSKAMYDAYWNGKFRDCAFHSGFNEDPFVCEYQGQSSDLPQPPVNAAAHHHHHH

Note: The underlined peptide corresponds to the PelB leading sequence that is processed during protein production and export to the periplasm.

**Simple model of conjugation**

The processes of growth and conjugation can be represented by the following chemical equations, using recipient cells (*F^–^*), donor cells (*F^+^*), and transconjugants (*F^+^*). The green color denotes yellow fluorescence in the experimental system.

(1)

(2)

(3)

(4)

(5)

If we assume that transconjugant donors grow and conjugate with the same rate constants as the original donors, we can further simplify equations (3) and (5) to obtain the following:

(3a)

(5a)

The corresponding differential equations are given below:

$\frac{d[F^{+}]}{dt}=k_{g,F^{+}}[F^{+}]$ (6)

$\frac{d[F^{+}]}{dt}=k_{g,F^{+}}[F^{+}]+k_{c}[F^{-}]([F^{+}]+[F^{+}])$ (7)

$\frac{d[F^{-}]}{dt}=k_{g,F^{-}}[F^{-}]-k_{c}[F^{-}]([F^{+}]+[F^{+}])$ (8)

In the case of equations (7) and (8), another simplification can be applied by assuming that the concentration of recipient cells is sufficiently high that the rate of conjugation would depend solely on the concentration of the donor cells. Then we have:

$\frac{d[F^{+}]}{dt}=k_{g,F^{+}}[F^{+}]+k_{c}([F^{+}]+[F^{+}])$ (7a)

$\frac{d[F^{-}]}{dt}=k_{g,F^{-}}[F^{-}]-k_{c}([F^{+}]+[F^{+}])$ (8a)

These equations can be solved analytically, applying the appropriate initial constraints to find the particular solutions. In our experiments, the concentration of transconjugants at time t=0 is zero, and the concentration of recipient cells at time t=0 is the initial concentration of recipient cells added to the mixture. The particular solutions are given by:

$[F^{+}]=[F^{+}_{0}]e^{k_{g,F^{+}}t}$ (9)

$[F^{+}]=-[F_{0}^{+}]e^{k_{g,F^{+}}t}+[F_{0}^{+}]e^{(k_{c}+k_{g,F^{+}})t}$ (10)

$[F^{-}]=-(\frac{k_{c}}{k_{c}+k_{g,F^{+}}-k_{g,F^{-}}})[F_{0}^{+}]e^{(k_{c}+k_{g,F^{+}})t}+([F_{0}^{-}]+(\frac{k_{c}}{k_{c}+k_{g,F^{+}}-k_{g,F^{-}}})[F_{0}^{+}])e^{k_{g,F^{-}}t}$ (11)

If we assume that the growth rates of the donors and recipients are equal, we can further simplify equation (11) to arrive at:

$[F^{-}]=-[F_{0}^{+}]e^{(k_{c}+k_{g,F^{+}})t}+([F_{0}^{-}]+[F_{0}^{+}])e^{k_{g,F^{+}}t}$ (12)

The proportion of F+ cells that were originally recipient cells is given by:

$\frac{[F^{+}]}{[F^{+}]+[F^{+}]}=1-e^{-k_{c}t}$ (13)

Because lags have been previously described (see main text), we also included a fitting parameter that could delay the onset of conjugation:

$\frac{[F^{+}]}{[F^{+}]+[F^{+}]}=1-e^{-k_{c}(t-t_{0})}$ (14)

The experimental data were fit to equation 14.

**Model for replicating phage and conjugation**

This model includes phage replication and infection in addition to conjugation. In this model there are six variables:

*x*, the concentration of F- cells (yellow),

*y*_0_, the concentration of cyan F+ cells that are uninfected with phage,

*w*_0_, the concentration of cyan F+ cells that are infected with phage,

*y*_c_, the concentration of yellow F+ cells that are uninfected with phage,

*w*_c_, the concentration of yellow F+ cells that are infected with phage, and

*v*, the concentration of free virus.

An F+ cell is yellow (subscript *c*) if either it or one of its predecessors was originally an F- cell that subsequently became an F+ cell after receiving the F plasmid via conjugation. An F+ cell that is cyan is thus one that was either in the original population of F+ cells or directly descended from a cell in that initial population.

The evolution in time of these variables is described by the following system of ordinary differential equations:

$$\dot{x}=aCx-D(v)\beta(x)(y_{o}+y_{c})-RD(v)\beta(x)(w_{o}+w_{c}),$$

$$\dot{y}_{o}=bCy_{o}-\gamma y_{o}v,$$

$$\dot{w}_{o}=cCw_{o}+\gamma y_{o}v,$$

$$\dot{y}_{c}=bCy_{c}+D(v)\beta(x)(y_{o}+y_{c})+RD(v)\beta(x)(w_{o}+w_{c})-\gamma y_{c}v,$$

$$\dot{w}_{c}=cCw_{c}+\gamma y_{c}v,$$

$$\dot{v}=d(w_{o}+w_{c})-ev.$$

The rates *a*, *b*, and *c* are the growth rates of the F-, uninfected F+, and infected F+ cells, respectively. The factor *C* ensures that the total cell population does not exceed a certain carrying capacity. If we write

$$C(x,y,w)=1-\frac{x+y_{o}+w_{o}+y_{c}+w_{c}}{K},$$

then the maximum population size is *K*. The existence of a carrying capacity reflects the limited volume and nutrients available in the culture. In our experiments, the saturation density *K* is ~ 4.4 x 10^8^ cells/mL.

Conjugation occurs when an F+ cell transmits the F plasmid to an F- cell via the pilus. The rate of such a ``bimolecular'' reaction would normally be written as *βxy*, where *β* is a constant, but experimental evidence indicates that conjugation essentially occurs at a constant rate (per unit concentration of F+ cells) when the concentration of F- cells is above a certain threshold (see main text). Therefore, we write the conjugation rate as *β*(*x*)*y*, where

$$\beta(x)=\left\{\begin{matrix} 0.4, & \text{if }x>8\times10^{6}, \\ \frac{0.4}{8\times10^{6}}x, & \text{if }x\leq8\times10^{6}. \end{matrix}\right.$$

In practice, our conjugation experiments are conducted in the regime where *β*=0.4 /hr. As discussed in a previous section of the supplement, when phage particles bind to an F+ cell's pilus, they inhibit the ability of the cell to transmit a copy of the plasmid to F- cells. Hence, the conjugation rate *β*(*x*) is reduced by a factor *D*(*v*) that is a function of the free phage concentration. Based on our data, we have

$$D(v)=\frac{1}{1+v/K_{d}},$$

where *K_d_* ~ 1.4 x 10^9^ particles/mL, or about 2 pM.

F+ cells that are infected are also slightly less effective at conjugation than F+ cells that are uninfected: infected cells conjugate at *R* times the rate of uninfected cells, where 0 < *R* < 1. Phage particles are produced by infected F+ cells at rate *d* and are degraded at rate *e*. They infect (uninfected) F+ cells at rate γ.

We now describe how we impute the parameters *d*, *e*, γ, and *R* given experimental values for all the other parameters. To determine the phage replication rate, our experimental data give the total concentration of F+ cells (*y*_0_ + *w*_0_) and the total concentration of phage (*v*) when beginning with a culture that consists entirely of pre-infected F+ cells and phage. The dynamics of this system do not depend on γ (since all cells are already infected) or *R* (since no conjugation is occurring). We then choose the *d* and *e* that minimize the sum of the squares of the errors between the model-generated time series of *y*_0_ + *w*_0_ and *v* and the experimental time series. For our data, we find that *d* = 62.53 and *e* = 0, indicating a phage production rate of about 60 particles per hour per infected cell. Also, very little phage degradation appears to occur, consistent with our general knowledge about phage storage and stability.

To determine *R* and γ, we have experimental time series for (A) the ratio of the concentration of citrine F+ cells to all F+ cells (when starting with a culture that has both F- and F+ cells and varying amounts of replicating phage) and (B) the concentrations of F+ cells and phage (again when starting with a culture that has F- and F+ cells and phage). Call the former data set {A} and the latter {B}. We first supposed that *R* = 1. Then we imputed the phage infectivity rate γ  using {B} (by choosing the γ  that minimizes the sum of the squares of the errors between the model and experimental timeseries). Using that value of γ, we imputed *R* using the data set {A}. Then we re-imputed γ  using this new value of *R* instead of *R* = 1. We continued this process iteratively until both *R* and γ no longer changed. For our data, we found that γ = 1.16 x 10^-8^ and *R* = 0.2.

The imputed parameters are those that minimize the sum of the squares (SS) of the errors between the model time series and the experimental time series. We can get a sense of how robust the imputation is by asking how much the imputed parameter varies as SS increases. For example, we find that the phage production rate *d* is 62.53; this is the value that minimizes SS. We denote this minimum value for SS as SS_0_. Now we can ask what possible values of *d* are such that the corresponding sum of the squares of the errors is no larger than 1.1(SS_0_) (a 10% error). For *d*, we obtain the interval [60.33, 64.73]. If we take the sum of the square of the errors as a measure of how good the fit is (smaller values are better), then this suggests that if we decrease the quality of the fit by 10%, then the range of possible values of *d* increases by approximately 3.5%.

The same analysis for *R* yields the range [0.14, 0.28]. The imputation of γ,  however, was not as robust as the imputation of *d* and *R*: its value can differ by several orders of magnitude (in both directions) without increasing the sum of the squares of the errors by more than 10%.
